# Alteration of Transthyretin Microheterogeneity in Serum of Multiple Trauma Patients

**Published:** 2007-08-08

**Authors:** Beate Gericke, Jens Raila, Maria Deja, Sascha Rohn, Bernd Donaubauer, Britta Nagl, Sophie Haebel, Florian J. Schweigert, Udo Kaisers

**Affiliations:** 1 Department of Physiology and Pathophysiology, Institute of Nutritional Science, University of Potsdam; 2 Department of Anaesthesiology and Intensive Care Medicine, Charité, Campus Virchow-Klinikum, Universitätsmedizin Berlin; 3 Department of Food Analysis, Institute of Food Technology and Food Chemistry, Technical University of Berlin; 4 Interdisciplinary Center for Mass Spectrometry of Biopolymers, University of Potsdam

**Keywords:** polytrauma, modification, microheterogeneity, TTR

## Abstract

Transthyretin (TTR) which exists in various isoforms, is a valid marker for acute phase response and subclinical malnutrition. The aim of the study was to investigate the relationship between inflammation, oxidative stress and the occurrence of changes in microheterogeneity of TTR.

A prospective, observational study at a level-I trauma center of a large urban medical university was performed. Patients were severely injured (n = 18; injury severity score (ISS): 34–66), and were observed within the first 24 hours of admittance and over the following days until day 20 after injury. 20 healthy subjects, matched by age and sex, were used as controls.

TTR was enriched by immunoprecipitation. Microheterogeneity of TTR was determined by linear matrix assisted laser desorption/ionization-time of flight-mass spectrometry (MALDI-TOF-MS). Four major mass signals were observed for TTR representing native, S-cysteinylated, S-cysteinglycinylated and S-glutathionylated TTR. In the course of their ICU stay, 14 of the 18 patients showed a transient change in microheterogeneity in favour of the S-cysteinglycinylated form of TTR (p < 0.05 vs. controls). The occurrence of this variant was not associated with the severity of trauma or the intensity of the acute-phase response, but was associated with oxidative stress as evidenced by Trolox.

Our results demonstrate that changes in microheterogeneity of TTR occur in a substantial number of ICU trauma patients. The diagnostic values of these changes remains to be elucidated. It is speculated that TTR modification may well be the mechanism underlying the morphological manifestation of amyloidose or Alzheimer’s diseases in patients surviving multiple trauma.

## Introduction

Transthyretin (TTR), formerly called prealbumin, belongs to the group of proteins including thyroxine-binding globulin and albumin, which bind to and transport thyroid hormones in the blood. TTR is also involved in the metabolism of vitamin A as it binds retinol-binding protein (RBP), the specific serum transport protein for retinol. First identified in 1942 by Kabat et al. in serum and cerebrospinal fluid, TTR has been described as a so-called visceral protein that is synthesized in the liver in response to nutritional supply. Therefore, TTR serum levels can be used as a sensitive biochemical parameter of subclinical malnutrition, since both the synthesis of proteins as well as energy intake are reflected in its serum levels. Serum levels of TTR are however also affected by acute and chronic diseases associated with an acute-phase response. Under these conditions, liver activity is concentrated on the synthesis of acute-phase response proteins, resulting in a drop in visceral proteins despite adequate nutritional supply (Ingenbleek and Young, 1994; Lasztity et al. 2002; Power et al. 2000; Abraham et al. 2003). Mutations in the TTR gene are the most common cause of autosomal dominant systemic amyloidosis such as the familial amyloid polyneuropathy (Quintas et al. 1999; Merlini and Bellotti, 2003). In these amyloidoses, the pathological deposits are characterized by the high abundance of TTR-β-structured-fibrils (Merlini and Bellotti, 2003). TTR has a single cysteine residue in position 10 that can exist in the SH form or as a mixed disulfide with the amino acid cysteine as well as with the peptides cysteinylglycine and glutathione (Bernstein and Ingenbleek, 2002; Schweigert et al. 2004). Additionally, Cys10 adducts of the S-homocysteine of TTR have been detected in serum of humans with hyperhomocysteinemia (Sass et al. 2003). It has recently been discussed that post-translational modifications of TTR represent a potential risk factor for the development of senile systemic amyloidosis (Zhang and Kelly, 2003). In-vitro TTR modified at the cys10 is found to be more susceptible to the formation of fibrils as precursors of amyloid deposits (Zhang and Kelly, 2003). Modifications of the TTR molecule might not only result in changes in its function but may also serve, for instance, as a biochemical marker for oxidative stress.

Free radical-derived reactive oxygen species (ROS) are constantly generated in living tissues, potentially damaging DNA, proteins and lipids. “Oxidative stress” occurs if ROS reaches abnormally high concentrations. Several pathological conditions such as atherosclerosis, carcinogenesis, neurodegenerative diseases, rheumatoid arthritis, and cataracts, as well as aging and cell death, have been linked to oxidative damage in various cell components (Berger, 2005). Antioxidative compounds, such as glutathione, play a protective role in minimizing the deleterious consequences of oxygen activation processes. Essentially, their beneficial effect occurs due to their ability to reduce disulfides or to oxidize themselves to disulfides. Once formed, thiol groups can also generate mixed disulfide bonds with protein thiol residues. Thus, besides the potential protection of protein-cysteine(Cys)-residues from irreversible oxidative modifications, thiol/disulfides can recruit protein thiol residues for the antioxidative buffering system (Tobaben et al. 2003).

Procedures which occur in connection with polytrauma induce a host defense response. This is characterized by local and systemic release of pro-inflammatory cytokines, arachidonic acid metabolites, contact phase and coagulation proteins, complement factors and acute phase proteins, as well as hormonal mediators. It is defined as systemic inflammatory response syndrome and is determined according to clinical parameters. However in parallel, anti-inflammatory mediators are produced (compensatory anti-inflammatory response syndrome) (Keel and Trentz, 2005). The production of free radicals is supported by several stress factors. Enzymatic protecting systems react to oxidative stress by positive adaptation. The non-enzymatic antioxidative systems (α-tocopherol, ascorbic acid, selen) are diminished, indicating an increased requirement (Kreinhoff et al. 1990).

Due to the fact that TTR acts as a negative acute-phase protein and based on the assumed interaction between changes in its mircroheterogeneity through the exchange at the Cys on position 10 and oxidative stress, we assumed a role for TTR modifications in acute phase reaction and oxidative stress in multiple trauma patients. Therefore, we investigated the microheterogeneity of TTR and its relation to systemic inflammation in severely trauma patients.

## Methods

### Patients

A total of 18 severely injured intensive care unit (ICU) patients were randomly selected and assessed within the first 24 hours after trauma and over the following days until day 20. The study protocol was approved by the ethic committee of the hospital and informed consent was obtained from legal substitutes. 20 healthy (based on physical and clinical-chemical parameters) subjects, matched by age and sex, were used as controls. The Injury Severity Score (ISS) is an anatomical scoring system whereby the score from the 3 most severely injured body regions (Head, Face, Chest, Abdomen, Extremities (including Pelvis), External) is squared and added together to produce the ISS score (ISS: 45 ± 7.5, mean ± SD). Blood samples were taken within the first 24 hours and at day 20 after injury.

### Immunoprecipitation of TTR and MALDI-TOF-MS

15 μl of serum was treated with equal amounts of a polyclonal rabbit anti human TTR or RBP (DakoCytomation, Denmark) and with 15 μl Sephadex G-15, 1 mg/ml phosphate buffer saline (PBS), (Pharmacia Fine Chemicals, Sweden). The mixture was incubated for two hours at 37 °C and then centrifuged at 15.000 × g for 15 min at room temperature. The supernatant was removed and the immunoprecipitated complex of TTR was extensively washed three times with PBS and then after final centrifugation, it was dissolved in 5 μl PBS. This was followed by a two step preparation procedure: Firstly, 1 μl immunoprecipitated sample was deposited on the target and dried. Secondly, 0.5 μl matrix (a saturated solution of sinapinic acid in acetonitrile plus water (1:2; v/v) containing 0.1% trifluoroacetic acid) was placed on the serum drop and also dried. This step was repeated.

MALDI mass spectra of the immunoprecipitated TTR was obtained using a Reflex II MALDI-TOF mass spectrometer (Bruker-Daltonik, Bremen, Germany). MALDI-TOF MS was performed in linear mode at 20k acceleration voltage. For ionisation, a nitrogen laser (VFL-337ND, LSI, MA, Newton, USA; 337 nm, 3 ns pulse width, 3 Hz) was used. The spectra were calculated by using external calibration with ions produced from horse cytochrome c (m/z 12,360.08) and horse myoglobin (m/z 16,951.46). After external calibration, this mass spectrometer was capable of achieving ~0.1% mass accuracy in the linear mode.

The samples were prepared in a two step procedure as follows. To determine the disulfide linkage of TTR adducts, the immunoprecipitated TTR was treated with dithiothreitol (DTT). DTT solution (100 mM) in buffer (100 mM NH_4_CO_3_, pH 8.8) was added to the solution at a ratio of 1:1 v/v (DTT solution volume/TTR solution volume). The mixture was incubated for 2 h at room temperature. Thereafter samples were immunoprecipitated as described and subsequently subjected to MALDI-TOF-MS.

### Quantitative analysis of C-reactive protein (CRP) and TTR

Serum TTR was quantitatively determined by an ELISA method using a polyclonal rabbit anti-human antibody (DakoCytomation, Hamburg, Germany). The TTR was measured by use of an ELISA technique adapted from the RBP procedure (Schweigert et al. 2003). In detail, wells of microtiter plates were coated through the addition to each well of rabbit anti-human TTR IgG (DakoCytomation, Hamburg, Germany) diluted 1:2000 in 50 μl of 50 mM carbonate buffer (pH 9.6) and then incubated for 2 hours at 37 °C. Plates were washed 4 times with PBS-Tween (pH 7.4), shaken dry, wrapped in plastic, and stored overnight at 4 °C. For analysis, nonspecific binding was blocked by the addition of 1% BSA diluted in PBS and incubated for 2 hours at 37 °C. After 4 additional washings, 50 μl of TTR standard (N Protein Standard/Standard SL OQIM 13, Dade Behring GmbH, Marburg, Germany) or serum sample diluted with PBS-Tween (pH 7.4) was placed in triplicate wells and incubated for 2 hours at 37 °C with constant shaking. After rinsing the wells with PBS-Tween (pH 7.4), 50 μl of peroxidase-conjugated sheep anti-human TTR IgG (Biotrend, Cologne, Germany), diluted 1:2000 in PBS-Tween with 1% BSA was added to each well, and plates were incubated for 1 hour at 37 °C. After 4 final washings, color was developed by the addition of *o*-phenylenediamine dihydrochloride solution (OPD, Sigma, Deisenhofen, Germany) and incubated for 20 minutes at 25 °C. OPD solution (100 μl/well) consisted of 3.7 mM solution in 50 mM disodium phosphate-25 mM citric acid buffer (pH 5.2) containing 0.012% H_2_O_2_. The reaction was stopped by the addition of 1 M H_2_SO_4_ (50 μl/well) and absorbance was measured at 490 nm by use of a spectrophotometer (Microplate Reader, Bio-Rad, Munich, Germany). The intra-assay coefficient of variation was 8.8%, and interassay coefficient of variation was 8.1%.

CRP levels in serum were measured with a high sensitivity latex turbimetric immunoassay using a latex-coupled monoclonal mouse anti-human antibody (Olympus AU 600, Biomed, Germany). The sensitivity of this assay was 0.005 mg/dl. The 90th percentile of normal CRP distribution was 0.3 mg/dl.

### Trolox equivalent antioxidant capacity (TEAC) assay

To measure the antioxidant capacity, the TEAC assay, described by Re et al. (Re et al. 1999), was used with minor modifications. This method is based on the reaction of the blue/green stable ABTS radical (ABTS*), which is formed through the reaction of ABTS and K_2_O_8_S_2_, with antioxidants. In this reaction, the blue/green color disappears because the ABTS* reacts with the antioxidant. This decolorization is determined spectrophotometrically at 734 nm after 6 min. The reduction in absorbance is related to that of Trolox, a synthetic, hydrophilic vitamin E analogue, which gives the TEAC value. The TEAC is calculated as millimoles of Trolox equivalents per litre.

## Analysis of Carotenoids and α-Tocopherol

For separation and quantification of carotenoids (α-carotene, β-carotene, lutein, zeaxanthin, canthaxanthin, β-cryptoxanthin, lycopene) and α-tocopherol, a gradient reversed-phase HPLC-system was used (Waters, Eschborn, Germany) as described in the literature. (Schweigert et al. 2003). Results of α-tocopherol and β-carotene were compared with the standard reference material 968a [National Institute of Standards Technology (NIST), Gaithersburg, MD, USA]. Carotenoid standards of lutein, zeaxanthin, canthaxanthin, β-cryptoxanthin and lycopene were a generous gift from Hoffman-La Roche, Switzerland and the standards for α-carotene, β-carotene and α-tocopherol were from Sigma (Deisenhofen, Germany).

### Statistical procedures

All data are given as a mean value ± SD. Statistical analysis was performed using nonparametric procedures. The Mann-Withney U-rank test was used to test for significant differences between patients and controls. The Wilcoxon test was used for the comparison between results of the first 24 hours and day 20 after injury. Values of p < 0.05 were considered significant.

## Results

Patients studied were predominantly male (age 32 ± 11 yrs) who had a mean Injury Severity Score (ISS) of 45.2 ± 7.5 (Table 1). Levels of TTR did not differ within the first 24 hours up to day 20 after trauma (4.0 ± 2.6 μmol/L vs. 4.1 ± 3.0 μmol/L; mean ± SD; n.s.). The CRP values tended to be lower on day 20 after injury (13.3 ± 8.4, 6.1 ± 4.6; mean ± SD; Table 2).

**Figure 1:**
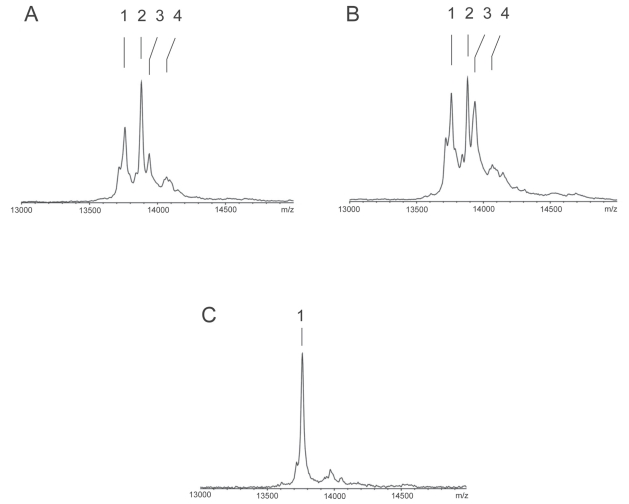
Representative mass spectra* of TTR in a healthy control (**IA**) and ICU patient after multiple trauma (**IB**). Control shows the native TTR (1 = 13,761 Da), the cysteinylated TTR (2 = 13,881 Da), the cysteinylglycinated TTR (3 = 13,937 Da) and the glutathionylated TTR (4 = 14,068 Da). In contrast, the serum TTR of a trauma patient (IB) shows a more dominant peak for the cysteinylglycinated TTR. Treatment with DTT (**IC**) resulted in a mass shift and native TTR remains the most dominant peak. *Molecular weights resulted from internal calibration.

Four dominant peaks were detected after immunoprecipitation by MALDI-TOF-MS (external calibration) in the range where TTR and its conjugated forms should normally appear (m/z 13,700–14,100), with a dominant peak at 13,878 ± 7 m/z (controls). The molecular mass of 13,757 ± 6 Da (controls) corresponded to the native, unmodified TTR. The other ion peaks showed molecular masses of 121 ± 3, 179 ± 3 and 298 ± 5 Da larger than native TTR, representing Cys^10^ adducts for S-cysteine (TTR-Cys^10^-S-S-Cys, mass = 13,877 ± 7 Da), S-cysteinylglycine (TTR-Cys^10^-S-S-CysGly, mass = 13,934 ± 6) and S-glutathione (TTR-Cys^10^-S-S-SG, mass = 14,056 ± 15) for controls. Figure 1 compares a representative mass spectrum obtained from a trauma patient within the first 24 hours after injury with the mass spectrum of a healthy individual. The treatment with DTT of the immunoprecipitated TTR from the same patient resulted in a loss of microheterogeneity and a shift towards the native form of TTR.

No differences in molecular masses of the individual signals were detected between controls and patients at different time points after injury, or between groups with or without modification. However a transient change in the microheterogeneity was observed. The extent of modification was defined on the basis of the relationship between peak height of the dominant cysTTR and the other variants as has been done previously (24, 26). In healthy persons the relation between cysTTR (100%) and cysglycTTR is 40%. A substantial change was thus defined if the extent of modification of cysglyc reached values twice as high as in healthy controls. We therefore used 80% as a cutoff. In 14 out of 18 multiple trauma patients a more dominant peak for the cysglycTTR (>80%) was present; this was again normalized at day 20 after hospitalization (Fig. 1). At this time point the TTR microheterogeneity pattern was comparable to healthy controls. Seven patients developed infection during their ICU stay and all of them showed an increased cysglycTTR modification, whilst none of those without a high cysglycTTR actually developed infection. The peak height ratio in patients within the first 24 hours after injury was significantly different to controls (Fig. 1). On day 20, no significant difference in peak height ratio was measured between patients and controls.

Trolox values were significantly lower in patients with the modification in TTR molecules in favor of the cysglyc form (3.1 mmol/L ± 0.4 versus 2.9 ± 0.3, p < 0.05); Trolox values were significantly higher on day 20 after injury. Serum α-tocopherol levels were significantly increased (p < 0.05) whilst for serum levels of carotenoids in total as well as for the individual carotenoids, except for β-carotene there was no difference between day 20 and the initial values at admission (Table 2).

## Discussion

The detection and characterization of variants and modified structures of proteins are clinically important for diagnosis purposes and for the elucidation of the pathogenesis of various diseases. Modern mass spectrometry methods have largely replaced analyses by conventional protein chemistry. MALDI-TOF-MS after immunoprecipitation allows the measurement of the molecular weight of intact TTR and the elucidation of the modified structures. Post-translational modifications of TTR have enormous diagnostic value. Some variant proteins cause diseases, and some diseases result in increase of proteins with abnormally modified structures. The protein TTR occurs in over 100 variants and most of them cause amyloidosis (6, 7). A recently published study reported on differential post-translational modifications of TTR in Alzheimer’s disease (26). The results of our study demonstrate the link between polytrauma and a clear change in microheterogeneity of TTR. Although the exact consequence on the stability of TTR and the long term outcome of polytrauma patients remains to be elucidated, modifications may influence the interaction of TTR with other proteins (RBP, Thyroxin), as well as having an effect on many aspects of the metabolism of the protein such as receptor binding, tissue uptake, degradation and excretion.

A negative correlation between levels of CRP and TTR in serum has already been shown (Gericke et al. 2005). This is supported by our data from multiple trauma patients at the two time points. Due to the still increased CRP levels at day 20 in comparison to healthy persons, a return of TTR to normal levels was also not observed (Table 2). In contrast, however, the amount of serum TTR in ICU patients on day 20 after injury differed significantly from healthy controls (Table 2). These quantitative differences are in accordance with the decrease in serum levels associated with TTR that has been previously reported (Clark et al. 1996).

Despite the fact that no quantitative changes in serum TTR were present in the patient population an obvious difference in the microheterogeneity of the protein was found. Our results support and confirm previous studies with regards to molecular variants of TTR in serum of humans (Terazaki et al. 1998; Kiernan et al. 2002; Schweigert et al. 2004). In all samples the cysTTR was dominant.

No significant difference in peak height ratio was found between patients and controls on day 20; however the modified cysglycTTR tended to remain elevated. This corresponds to the still elevated serum levels of CRP and the still decreased serum levels of TTR. Interestingly, Trolox values were significantly different in patients with the modification in TTR molecules in favor of the cysglyc form, suggesting that oxidative stress might contribute to this specific microheterogeneity of TTR. Although Trolox values were significantly higher on day 20 after injury, indicating an improvement in oxidative status, serum antioxidants showed contradictory changes. While the increased α-tocopherol levels are comparable with those found in healthy persons, carotenoid levels were substantially lower than in healthy well nourished individuals (Schweigert et al. 2003). Similar low levels have previously been observed in critically ill individuals (Weiss et al. 1998). Lower serum levels of carotenoids have been demonstrated to be associated with a reduced dietary uptake or metabolic change in consequence of the acute phase response (Schweigert, 2001). Both aspects have to be considered with regard to the results of this study. In ICU patients receiving parenteral nutrition only α-tocopherol is routinely supplemented. Therefore, it can be assumed that tocopherol might have contributed to the slight improvement of antioxidative status at the end of this study.

Modifications in the microheterogeneity of TTR have been associated with oxidative stress (Kishikawa et al. 2002; Tajiri et al. 2002). The improvement of high-throughput analysis for these modifications has created increasing interest in the diagnostic relevance of such modifications in cases such as intoxications with molybdenum or the occurrence of protein modification through homocysteine (Sass et al. 2003). Recently, the ratio of native to cysteinylated TTR in cerebrospinal fluid has been suggested as a useful diagnostic tool for Alzheimer’s disease (Biroccio et al. 2006). In our patients, the cysglycTTR was associated with sepsis and was related to the antioxidative status.

Based on numerous studies, it can be assumed that the transient modifications in the microheterogeneity of TTR in our patients were of functional consequence. Most importantly, it has been demonstrated that such modifications influence the amyloidogenicity of TTR (Kishikawa et al. 1999, Lim et al. 2003b). TTR is an important constituent among the more than 20 proteins and peptides identified in different amyloid lesions (Westermark et al. 1999). Alzheimer’s disease is characterized by two pathological hallmarks: extracellular- or senile amyloid plaques generated by the deposition of insoluble amyloid fibrils (Serpell, 2000) and intracellular neurofibrillary tangles (Bossy-Wetzel et al. 2004). These deposits are caused by accumulation of misfolded autologous proteins in tissues or organs (Merlini and Bellotti, 2003). With regards to TTR, the formation of TTR amyloid deposits is associated with the destabilization of the homotetramer complex which is either modified by small molecules interacting with the central binding site for thyroxin (Olofsson et al. 2001), or by the post-translational modification at the Cys10 site of the molecule. This modification, which possibly has a genetic cause, changes the three-dimensional structure and destabilizes the complex (Zhang and Kelly, 2003). Modifications such as the S-sulfonation and S-thiolation or an increase in doubly oxidized TTR were reported and had been linked to modifications of TTR as well as amyloid formation (Lim et al. 2003a) (Heegaard et al. 2006). At this stage it remains speculative, as to whether short term changes in the TTR microheterogeneity may be indicative of an individual susceptibility to destabilizing modifications in TTR, or whether transient changes might be of long term consequence for the affected ICU patient with regard to amyloidosis, Alzheimer’s disease, or the occurrence of other neurological disorders.

## Conclusion

In conclusion, our results demonstrate that trauma associated transient changes in the microheterogeneity of TTR occurred in 78% of our ICU patients. This modification was characterized by a transient abundance of the mixed disulfide cysglycTTR. The context in which these changes occur, the extent to which such changes can be used for diagnostic purposes, as well as their role as a possible risk factor for the development of amyloidosis, Alzheimer’s disease or other diseases remains to be elucidated. It was, however, possible to demonstrate a link between the modulation of a multifunctional protein, inflammation and the response after injury.

## Figures and Tables

**Table 1:** General characteristics of ICU patients (n = 18) at day of admission.

Age (years)	32.2 ± 11.6
Sex	3 female/15 male
Height (cm)	179.3 ± 11.6
Weight (kg)	85.0 ± 10.7
Injury Severity Score (ISS)	45.2 ± 7.5
Abbreviated Injury Scale (AIS)	13.1 ± 2.1
Acute Physiology And Chronic	16.5 ± 8.2
Health Evaluation (APACHE II) Simplified Acute Physiology	27.1 ± 9.5
Score (SAPS II) first 24 hours SAPS II day 20	13.9 ± 7.9
Sepsis-related Organ Failure	7.9 ± 3.1
Assessment (SOFA) first 24 hours	
SOFA day 20	1.7 ± 2.1

**Table 2:** The most important parameters used to characterize the antioxidative status and the post trauma response of ICU patients.

	First 24 hours	Day 20 after injury	Modification on cysGlysTTR	Without changes in modification
Trolox (mmol/L)	2.8 ± 0.4^a^	3.1 ± 0.4	3.1 ± 0.4	2.9 ± 0.3
α-tocopherol (μmol/L)	17.4 ± 5.5^b^	22.3 ± 6.7	19.5 ± 7.3	19.3 ± 6.9
α-carotene (μmol/L)	0.04 ± 0.02	0.04 ± 0.03	0.03 ± 0.02	0.04 ± 0.03
β-carotene (μmol/L)	0.22 ± 0.10	0.23 ± 0.20	0.21 ± 0.14	0.20 ± 0.11
Lutein (μmol/L)	0.06 ± 0.02	0.04 ± 0.03	0.05 ± 0.03	0.04 ± 0.02
Zeaxanthin (μmol/L)	0.006 ± 0.002	0.004 ± 0.004	0.005 ± 0.003	0.004 ± 0.002
Canthaxanthin (μmol/L)	0.013 ± 0.005	0.006 ± 0.003	0.011 ± 0.006	0.010 ± 0.005
β-cryptoxanthin (μmol/L)	0.06 ± 0.03	0.04 ± 0.05	0.05 ± 0.03	0.04 ± 0.02
Lycopene (μmol/L)	0.06 ± 0.03	0.04 ± 0.02	0.05 ± 0.03	0.05 ± 0.03
CRP (mg/dL)	13.3 ± 8.4	6.1 ± 4.6	13.3 ± 9.0	12.3 ± 7.0
Total carotenoids (μmol/L)	0.5 ± 0.2	0.4 ± 0.3	0.4 ± 0.2	0.4 ± 0.2
TTR (μmol/L)	4.0 ± 2.6	4.1 ± 3.0	4.7 ± 3.4	3.5 ± 2.7

a Trolox is significantly different from patients with or without modification (p < 0.05) and between both time points (p < 0.05).

b α-tocopherol tends to be different between patient within the first 24 hours and on day 20 after injury.
